# Robust gene selection methods using weighting schemes for microarray data analysis

**DOI:** 10.1186/s12859-017-1810-x

**Published:** 2017-09-02

**Authors:** Suyeon Kang, Jongwoo Song

**Affiliations:** Department of Statistics, Ewha Womans University, Seoul, South Korea

**Keywords:** Microarray data, Gene selection method, Significance analysis of microarrays, Noisy data, Robustness, False discovery rate

## Abstract

**Background:**

A common task in microarray data analysis is to identify informative genes that are differentially expressed between two different states. Owing to the high-dimensional nature of microarray data, identification of significant genes has been essential in analyzing the data. However, the performances of many gene selection techniques are highly dependent on the experimental conditions, such as the presence of measurement error or a limited number of sample replicates.

**Results:**

We have proposed new filter-based gene selection techniques, by applying a simple modification to significance analysis of microarrays (SAM). To prove the effectiveness of the proposed method, we considered a series of synthetic datasets with different noise levels and sample sizes along with two real datasets. The following findings were made. First, our proposed methods outperform conventional methods for all simulation set-ups. In particular, our methods are much better when the given data are noisy and sample size is small. They showed relatively robust performance regardless of noise level and sample size, whereas the performance of SAM became significantly worse as the noise level became high or sample size decreased. When sufficient sample replicates were available, SAM and our methods showed similar performance. Finally, our proposed methods are competitive with traditional methods in classification tasks for microarrays.

**Conclusions:**

The results of simulation study and real data analysis have demonstrated that our proposed methods are effective for detecting significant genes and classification tasks, especially when the given data are noisy or have few sample replicates. By employing weighting schemes, we can obtain robust and reliable results for microarray data analysis.

**Electronic supplementary material:**

The online version of this article (10.1186/s12859-017-1810-x) contains supplementary material, which is available to authorized users.

## Background

Microarray technologies allow us to measure the expression levels of thousands of genes simultaneously. Analysis on such high-throughput data is not new, but it is still useful for statistical testing, which is a crucial part of transcriptomic research. A common task in microarray data analysis is to detect genes that are differentially expressed between experimental conditions or biological phenotype. For example, this can involve a comparison of gene expression between treated and untreated samples, or normal and cancer tissue samples. Despite the rapid change of technology and the affordable cost for conducting whole-genome expression experiments, many past and recent studies still have relatively few sample replicates in each group, which makes it difficult to use typical statistical testing methods. These two problems, high dimensionality and small sample size problems, have triggered developments of feature selection in transcriptome data analysis [1–9]. These feature selection methods can be mainly classified into four categories depending on how they are combined with learning algorithms in classification tasks: filter, wrapper, embedded, and hybrid methods. For details and the corresponding examples of these methods, we refer the reader to several review papers [10–18]. As many researchers commented, filter methods have been dominant over the past decades due to its strong advantages, although they are the earliest in the literature [11–13, 15, 16]. They are preferred by biology and molecular domain experts as the results generated by feature ranking techniques are intuitive and easy to understand. Moreover, they are very efficient because they require short computation time. As they are independent of learning algorithms, they can give general solutions for any classifier [15]. They also have a better generalization property as the bias in the feature selection and that of the classifier are uncorrelated [19]. Inspired by its advantages, we focus on the filter method in this study.

One of the most widely used filter-based test methods is significance analysis of microarrays (SAM) [1]. It identifies genes with a statistically significant difference in expression between different groups by implementing gene-specific modified *t*-tests. In microarray experiments, some genes have small variance so their test statistics become large, even though the difference between the expression levels of two groups is small. SAM prevents those genes from being identified as statistically significant by adding a small positive constant to the denominator of the test statistic. This is a simple but powerful modification for detecting differentially expressed genes, considering the characteristics of microarray data. Since its establishment, the SAM program has been repeatedly updated. The latest version is 5.0 [20].

We also aim to develop methods for detecting significant genes based on a deeper understanding of microarray data. Even when researchers monitor an experimental process and control other factors that might have an influence on the experiment, biological or technical error can still arise in high-throughput experiments. For example, when one sample among a number of replicated samples gives an outlying result owing to a technical problem, variance of the gene expression becomes larger than expected and its test statistic becomes small. This is a major issue because it can lead to biologically informative genes failing to be identified as having a significant effect. Therefore, we here attempt to reduce this increase in variance for such cases by modifying the variance structure of SAM statistics, using two weighting schemes. It is also important to adjust the significance level of tests. Since we generally need to test thousands of genes simultaneously, the multiple testing problem arises. To resolve this problem, several methods have been suggested as replacements for the simple *p*-value; for example, we can use the family-wise error rate (FWER), false discovery rate (FDR) [1, 21], and positive false discovery rate (pFDR) [22]. Among them, FDR, which is the expected proportion of false positives among all significant tests, is a popular method to adjust the significance level. It can be computed by permutation of the original dataset. The test procedures we propose in this paper also use FDR, the same as SAM.

Once a list of significant genes is established by a gene selection method, researchers may carry out further experiments such as real-time polymerase chain reaction to determine whether these reference genes are biologically meaningful. However, many genes may not be tested owing to limitations of time and resources. For example, even if hundreds of genes are included in a list of reference genes for a user-defined significance cutoff, researchers may just select a few top-ranked genes among them for further analyses. Therefore, it is very important that the genes are properly ranked in terms of their significance, especially for top-ranked genes [23, 24]. As such, in this paper, we focus on improving test statistics for each gene and assessing how well each test method identifies significant genes.

For microarray data analysis, a comparison of the performance of gene selection methods is difficult because we generally do not know the “gold standard” reference genes in actual experiments. In other words, we do not know which genes are truly significant. This is a common problem encountered in transcriptome data analysis, so most studies have focused on comparing classification performances, which are determined by the combination of the feature selection and learning algorithm. As these results are clearly dependent on the performance of learning method, we cannot compare the effectiveness of feature selection techniques definitively [16]. Therefore, in this paper, we generate spike-in synthetic data that allow us to determine which genes are truly differentially expressed between two groups. For this, we suggest a data generation method based on the procedure proposed by Dembélé [25]. By performing such simulations, we can see how the performance changes depending on the characteristics of the dataset, such as sample size, the proportion of differentially expressed genes, and noise level. In this study, we focus on comparing performance according to noise level as our goal is to efficiently detect significant genes in a noisy dataset. To verify that our proposed methods can also compete with previous methods for actual microarray data, we use two sets of actual data that have a list of gold standard genes based on previous findings. All of these real datasets are publicly available and can be downloaded from a website [26] and R package [27]. In order to compare different gene selection methods, we also define two performance metrics that can be used when true differentially expressed genes are known.

This paper is organized as follows. In the next section, we review the algorithm of SAM and propose statistical tests for microarray data that are modified versions of SAM, named MSAM1 and MSAM2. In addition, we explain our synthetic data generation method and suggest two performance metrics. In the results section, we describe our simulation studies and real data analysis. We compare SAM, MSAM1, and MSAM2 using 14 types of simulated dataset, which have different noise levels and sample sizes, and two sets of real microarray data. We next discuss the difference between the three methods in detail, focusing on FDR estimation. Additionally, we give the results of classification analysis using some top-ranked genes selected by each method. In the last section, we summarize and conclude this paper.

## Methods

In this section, we briefly review the SAM algorithm [1] and propose new modified versions of SAM, focusing on calculating the test statistic.

### SAM

Let *x*_*ij*_ and *y*_*ij*_ be the expression levels of gene *i* in the *j*th replicate sample in states 1 and 2, respectively. For such a two-class case, the states of samples indicate different experimental conditions, such as control and treatment groups. Let *n*_1_ and *n*_2_ be the numbers of samples in these two groups, respectively. The SAM statistic proposed in [1] is defined as follows:

$$ {d}_i=\frac{{\overline{x}}_i-{\overline{y}}_i}{s_i+{s}_0} $$

where ${\overline{x}}_i$ and ${\overline{y}}_i$ are the mean expression of the *i*th gene for each group,${\overline{x}}_i={\sum}_{j=1}^{n_1}{x}_{ij}/{n}_1$ and ${\overline{y}}_i={\sum}_{j=1}^{n_2}{y}_{ij}/{n}_2$. The gene-specific scatter *s*_*i*_ is defined as:

$$ {s}_i=\sqrt{a\left\{\sum_{j=1}^{n_1}{\left({x}_{ij}-{\overline{x}}_i\right)}^2+\sum_{j=1}^{n_2}{\left({y}_{ij}-{\overline{y}}_i\right)}^2\right\}} $$

where *a* = (1/*n*_1_ + 1/*n*_2_)/(*n*_1_ + *n*_2_ − 2) and *s*_0_ is a small positive constant called the fudge factor, which is chosen to minimize the coefficient of variation of *d*_*i*_. The computation of *s*_0_ is explained in detail in [3].

Now let us consider the overall algorithm. The SAM algorithm proposed in [1] can be stated as follows:

1. Calculate test statistic *d*_*i*_ using the original dataset.
2. Make a permuted dataset by fixing the gene expression data and shuffling the group labels under the *H*_0_ where *H*_0_: ${\overline{x}}_i-{\overline{y}}_i=0$ for all *i*.
3. Compute test statistics ${d}_i^{\ast }$ using the permuted data and order them according to their magnitudes as ${d}_{(1)}^{\ast}\le {d}_{(2)}^{\ast}\le \cdots \le {d}_{(n)}^{\ast }$, where *n* is the number of genes.
4. Repeat steps 2 and 3 *B* times and obtain ${d}_{(1)}^{\ast }(b)\le {d}_{(2)}^{\ast }(b)\le \cdots \le {d}_{(n)}^{\ast }(b)$ for *b* = 1 , 2 , … , *B*, where *B* denotes the total number of permutations.
5. Calculate the expected score ${d}_{(i)}^E={\sum}_{b=1}^B{d}_{(i)}^{\ast }(b)/B$.
6. Sort the original statistic from step 1, *d*_(1)_ ≤ *d*_(2)_ ≤  ⋯  ≤ *d*_(*n*)_.
7. For user-specific cutoff ∆, genes that satisfy $\mid {d}_{(i)}-{d}_{(i)}^E\mid >\Delta$ are declared significant. A gene is defined as being significantly induced if ${d}_{(i)}-{d}_{(i)}^E>\Delta$ and significantly suppressed if ${d}_{(i)}-{d}_{(i)}^E<-\Delta$.
8. Define *d*_(up)_ as the smallest *d*_(*i*)_ among significantly induced genes and *d*_(down)_ as the largest *d*_(*i*)_ among significantly suppressed genes.
9. The false discovery rate (FDR) is defined as the proportion of falsely significant genes among genes considered to be significant and can be estimated as follows:

$$ \widehat{\mathrm{FDR}}=\frac{\sum_{b=1}^B\#\left\{i:{d}_{(i)}(b)\ge {d}_{\left(\mathrm{up}\right)}\vee {d}_{(i)}(b)\le {d}_{\left(\mathrm{down}\right)}\right\}/B}{\#\left\{i:{d}_{(i)}\ge {d}_{\left(\mathrm{up}\right)}\vee {d}_{(i)}\le {d}_{\left(\mathrm{down}\right)}\right\}} $$

The algorithm consists of two parts: computation of the test statistic and determination of the cutoff for a given ∆. We will focus on the first of these parts and apply a simple modification to the computation of gene-specific scatter *s*_*i*_ to find a more robust test statistic. The numerator of the modified statistic and that of the original SAM statistic are the same. All of the procedures can be implemented using the *samr* package for Bioconductor in R. [20] described how to use the package and provided technical details of the SAM procedure.

### Modified SAM

From one experiment [28], we observed several cases in which most of the results of gene expression are very close to each other, apart from one substantial outlier. As a result, the ranks of these genes from SAM are lower than expected. This prompted us to propose a new test method that has a different variance structure, leading to robustness on identifying informative genes in the presence of outliers. Throughout the paper, we use the term “outliers” to indicate “unusual observations”.

Let us consider two cases with the following data: case 1: (5,5,5,5,8.54) and case 2: (3,4,5,6,7). For these two cases, the variance is the same, inferring that they have the same spread. However, even though the levels of variance are equal, in fact, we cannot say that the data points are similarly distributed. We believe that case 1 is more reliable than case 2. Our goal, therefore, is to propose a test statistic that has a more significant result for case 1 than for case 2. To minimize the effects of outliers among samples, we use the median instead of the mean and employ a weight function *w* when computing the test statistic, resulting in a less weight on an outlier sample that is far from other samples. A modified *s*_*i*_, ${\overset{\sim }{s}}_i$, is defined as follows:

$$ {\overset{\sim }{s}}_i=\sqrt{\sum_{j=1}^{n_1}w\left({x}_{ij}\right){\left({x}_{ij}-{median}_j\left({x}_{ij}\right)\right)}^2+\sum_{j=1}^{n_2}w\left({y}_{ij}\right){\left({y}_{ij}-{median}_j\left({y}_{ij}\right)\right)}^2} $$

Accordingly, our test statistic ${\overset{\sim }{d}}_i$ is defined as follows:

$$ {\overset{\sim }{d}}_i=\frac{{\overline{x}}_i-{\overline{y}}_i}{{\overset{\sim }{s}}_i+{s}_0} $$

Methods modified by this approach might be particularly useful when detecting differentially expressed genes from noisy microarray data. The key idea is to reduce the impact of outliers when calculating the test statistic. We propose two different weight functions in this paper. The values of ${\overset{\sim }{s}}_i$ and ${\overset{\sim }{d}}_i$ would differ quite markedly depending on the used weight function.

#### Modified SAM1 (Gaussian weighted SAM)

**Fig. 1:**
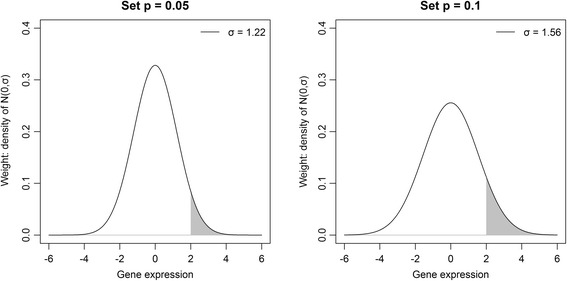
Two examples of the weight function for MSAM1 when ***m*** is 2. When setting ***p*** = 0.05, ***σ*** is determined to be 1.22 (left panel), and when setting ***p*** = 0.1, it is determined to be 1.56 (right panel). Since ***m*** is the 100(1 − ***p***)th percentile point of ***N***(0, ***σ***), the grey-shaded area in each panel is 0.05 and 0.1, respectively

The weight function used in Modified SAM1 (MSAM1) is based on the Gaussian kernel, which is a widely used weight that decreases smoothly to 0 with increasing distance from the center. It is defined as follows:

$$ w\left({x}_{ij};{\mu}_i,\sigma \right)=\frac{1}{\sigma}\phi \left(\frac{x_{ij}-{\mu}_i}{\sigma}\right) $$

where *ϕ* is the probability density function of a standard normal distribution, $\phi (x)={e}^{-{x}^2/2}/\sqrt{2\pi }$. The mean *μ*_*i*_ is a gene-specific parameter such that *μ*_*i*_ = median_*j*_(*x*_*ij*_) and standard deviation *σ* is a data-dependent constant determined by the following procedure: first, *m* is defined as follows. *m* = max(|*x*_*ij*_ − median_*j*_(*x*_*ij*_)|, |*y*_*ij*_ − median_*j*_(*y*_*ij*_)|). It is calculated from given data. Second, *p* is a user-defined value between 0 and 1. Finally, given *m* and *p*, we can find the value of *σ* that satisfies the following equation:

$$ m={F}^{-1}\left(1-p;0,\sigma \right) $$

where *F* is the cumulative distribution function of a normal distribution. Therefore, *m* would approximately be the 100(1 − *p*)th percentile point of a normal distribution with mean 0 and standard deviation *σ*. As can be seen from Fig. 1, smaller *p* yields smaller *σ*. Therefore, smaller *p* makes the weight applied to outlier samples smaller. On the other hand, as *p* increases, the results of original and modified SAMs become similar because the weight on the outlier is very similar to the weight on the non-outliers. In this research, we set *p* = 0.001 since we found that this value is sufficiently small to reduce the effect of outliers.

**Fig. 2:**
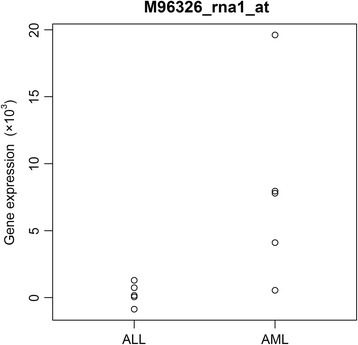
Gene expressions of M96326_rna1_at (Azurocidin) from 5 ALL patients and 5 AML patients

**Fig. 3:**
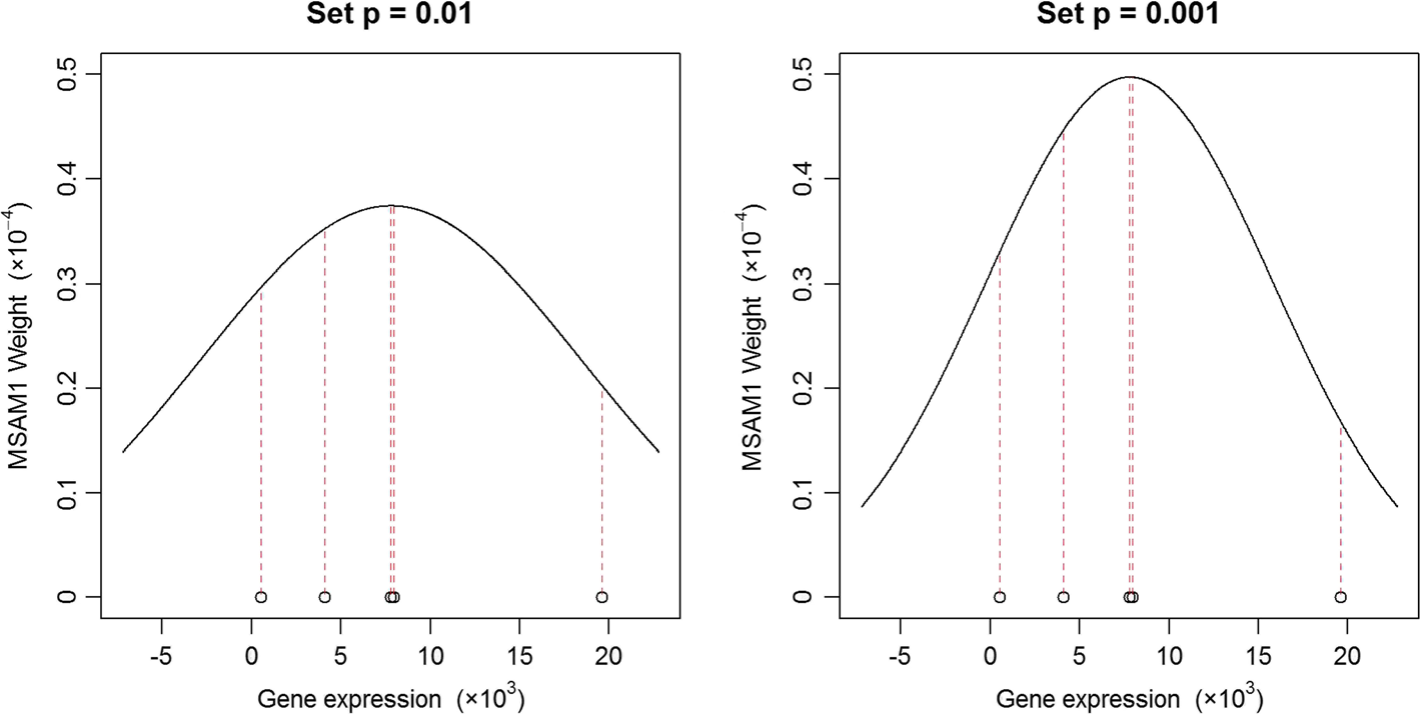
The left panel illustrates the weights of MSAM1 when *p* is 0.01. The right panel is the case when *p* is 0.001. In each panel, 5 black circle points are gene expressions of M96326_rna1_at (Azurocidin) from 5 AML patients. The lengths of 5 red dashed lines indicate the weights on the 5 observations

For a better understanding of MSAM1, we here illustrate the weight function of MSAM1 and its application in detail. Let us consider Leukemia data [29]; for details of this data, see real data analysis section. The data consist of 38 samples (27 from ALL patients and 11 from AML patients) and 7129 genes. For simplicity and clarity, we randomly selected five samples for each sample type and applied SAM, MSAM1 with *p* = 0.01 and MSAM1 with *p* = 0.001. In order to compare weights given by each method, let us take one gene, M96326_rna1_at (Azurocidin). This gene would be a good example to clarify the difference between SAM and MSAM1 because it has an outlier sample. From Fig. 2, we can see that gene expressions in group 1 are similar. On the other hand, one of five samples in group 2 is clearly far from others. Table 1 and Fig. 3 show its gene expressions and weights computed by SAM and MSAM1. In Fig. 3, the lengths of 5 red dashed lines indicate the weights on the 5 observations. As we stated above, we can also see that smaller *p* makes the difference between weights applied to outlier and non-outlier samples greater.

**Table 1:** Comparison of SAM and MSAM1 weights: an informative gene from leukemia data, M96326_rna1_at (Azurocidin)

	ALL					AML				
Gene expressions (×10^3^)	−0.86	0.05	0.16	0.74	1.30	0.55	4.11	7.79	7.96	19.60
SAM weights	1.00	1.00	1.00	1.00	1.00	1.00	1.00	1.00	1.00	1.00
MSAM1 weights (×10^−4^) for *p* = 0.01	0.37	0.37	0.37	0.37	0.37	0.30	0.35	0.37	0.37	0.20
MSAM1 weights (×10^−4^) for *p* = 0.001	0.49	0.50	0.50	0.50	0.49	0.33	0.45	0.50	0.50	0.17

#### Modified SAM2 (inverse distance weighted SAM)

This method uses Euclidean distance among the observations. The weight function used in Modified SAM2 (MSAM2) is defined as follows:

$$ w\left({x}_{ij}\right)=\frac{1}{\sum_k{d}_E\left({x}_{ij},{x}_{ik}\right)} $$

where *d*_*E*_(*x*_*ij*_, *x*_*ik*_) is the Euclidean distance between the *j*th and *k*th samples of gene *i*. The reason that we use this weight function can be explained by the following example. Let us assume that there are 10,000 genes (*i* = 1 , 2 , … , 10000). Also, suppose there are 4 sample replicates (observations) in a group of the first gene (*i* = 1) and their gene expressions are *x*_11_ , *x*_12_ , *x*_13_ and *x*_14_. Let *w*_*j*_ be the weight on *j*th observation for *j*=1, 2, 3 and 4. In this case, the weights on these observations are as follows.

$$ {w}_1={\left(\sum_{k=1}^4{d}_E\left({x}_{11},{x}_{1k}\right)\right)}^{-1},\kern0.75em {w}_2={\left(\sum_{k=1}^4{d}_E\left({x}_{13},{x}_{1k}\right)\right)}^{-1}, $$

$$ {w}_3={\left(\sum_{k=1}^4{d}_E\left({x}_{13},{x}_{1k}\right)\right)}^{-1},\kern0.75em {w}_4={\left(\sum_{k=1}^4{d}_E\left({x}_{14},{x}_{1k}\right)\right)}^{-1} $$

If *x*_11_ , *x*_12_ and *x*_13_ are close to each other and *x*_14_ is far from these 3 values, *w*_4_ is much smaller than *w*_1_ , *w*_2_ and *w*_3_. Therefore, by using this weight function, we can give a smaller weight to an outlier. The further away an observation is from the others, the smaller weight is given.

### Synthetic data generation

To run experiments, we need to generate synthetic gene expression data. These datasets should have characteristics similar to those of real microarray data to ensure that the results are reliable and valid. Two important characteristics of gene expression data, which are reported elsewhere [25, 30, 31] and also considered in this study, are as follows:

1. Under similar biological conditions, the level of gene expression varies around an average value. In rare cases, technical problems would result in values far away from this average.
2. Genes at low levels of expression have a low signal-to-noise ratio.

The ‘technical problems’ mentioned in the first of these points are one possible explanation for outliers observed in microarray data. Since our goal is to develop methods that detect differentially expressed genes well in a noisy dataset containing outliers, we consider not only a dataset with little noise, but also a noisy dataset with outliers. We ensure that outliers are present at higher probability in several of the datasets to provide a wider range of comparisons among the different test methods. Basically, we follow the microarray data generation model by Dembélé [25], which uses a beta distribution. In this article, we employ a beta and a normal distribution to generate data points, assuming that the levels of gene expression essentially follow such distributions. To allow outliers in generated data, we add a technical error term in our model; this term is mentioned in [25], but not used in their model. According to the noise level and distribution type, we consider four different simulation set-ups as follows: Scenario 1, non-contaminated beta; 2, contaminated beta; 3, non-contaminated normal; 4, contaminated normal. Therefore, data used in scenarios 1 and 3 have low noise level, and data used in scenarios 2 and 4 have high noise level. The step-by-step procedure for our data generation method is summarized as follows.

Step 1. Let *n* be the number of genes and *n*_1_ and *n*_2_ be control and treatment sample sizes, respectively.

Step 2. Generate *z*_*i*_ from a beta (normal) distribution for *i* = 1 , 2 , … , *n* and transform the values, ${\overline{z}}_i= lb+ ub\times {z}_i$.

Step 3. For each ${\overline{z}}_i$, generate (*n*_1_ + *n*_2_) values as follows: ${z}_{ij}\sim \mathrm{unif}\left(\left(1-{\alpha}_i\right){\overline{z}}_i,\left(1+{\alpha}_i\right){\overline{z}}_i\right)$, where ${\alpha}_i={\lambda}_1{e}^{-{\lambda}_1{\overline{z}}_i}$.

Step 4. The final model is given by

$$ {d}_{ij}={z}_{ij}+{s}_{ij}+{n}_{ij}+{t}_{ij} $$

where the term *s*_*ij*_ allows us to define differentially expressed genes. Their values are zero for the control group, ${s}_{ij}\sim N\left({\mu}_{de},{\sigma}_{de}^2\right)$ for genes with induced expression, and ${s}_{ij}\sim N\left(-{\mu}_{de},{\sigma}_{de}^2\right)$ for genes with suppressed expression, where ${\mu}_{de}={\mu}_{de}^{min}+\mathrm{Exp}\left({\lambda}_2\right)$. *n*_*ij*_ is an additive noise term, ${n}_{ij}\sim N\left(0,{\sigma}_n^2\right)$. The final term *t*_*ij*_ is used to define outlying samples by allowing non-zero values for some genes. The undefined parameters for each step can be set by the users. The values we use in this paper are as follows: *λ*_1_ = 0.13, *λ*_2_ = 2, ${\mu}_{de}^{min}=0.5$, *σ*_*de*_ = 0.5, *σ*_*n*_ = 0.4. For these parameters, the influence of different parameter settings on the generated data is well explained elsewhere [25].

#### Scenario 1: Beta with low noise level

In this case, we generate data points from Beta(*shape*_1_, *shape*_2_). *shape*_1_ and *shape*_2_ are two shape parameters of the beta distribution and we here set *shape*_1_ = 2 and *shape*_2_ = 4. We also set *lb* = 4, *ub* = 14. The values of *t*_*ij*_ are zero for this case.

#### Scenario 2: Beta with high noise level

Here, we generate a noisier data than above data. The generation procedure is basically the same as the above case, except for allowing some non-zero *t*_*ij*_. To make outlying samples, we contaminate the data by adding gaussian noise to some treatment samples: For genes with induced or suppressed expression,

$$ {t}_{ij}\sim N\left(0,{\sigma}_{\mathrm{deo}}^2\right) $$ for *j* = (*n*_1_ + *n*_2_ − *n*_deo_ + 1) , … , (*n*_1_ + *n*_2._)

where *σ*_deo_ is a non-zero constant and *n*_deo_ is the number of outlying samples. We here set *σ*_deo_ = 1 and *n*_deo_ = [0.2 × *n*_2_] where [*x*] = *m* if *m* ≤ *x* < *m* + 1 for all integer *m*. For example, if there are five sample replicates in a treatment group, there can be one possible candidate as an outlier. Therefore, *σ*_deo_ and *n*_deo_ control the distribution and noise level of outlying samples. We believe that this set-up is reasonable because it does not destroy the original data structure while controlling the noise level of the data.

#### Scenario 3: Normal with low noise level

This scenario assumes that the levels of gene expression essentially follow a normal distribution, instead of a beta distribution. In this research, we use the normal distribution with mean 10 and standard deviation 1.5 for generated data points to be distributed between realistic bounds; the gene expression levels on a log2 scale after robust multichip analysis normalization usually vary between 0 and 20. We set *lb* = 0, *ub* = 1 in Step 2, which means that no transformation is applied.

#### Scenario 4: Normal with high noise level

To generate a noisier normal data, we use the same data generation procedure of Scenario 3, except for allowing some non-zero *t*_*ij*_ in Step 4. The structure of *t*_*ij*_ is the same as in Scenario 2.

### Performance metrics

To compare the performance of several methods, we need several evaluation measures. Since we know which genes are differentially expressed in our simulated datasets, we can define two performance metrics as follows, measuring how well each method identifies these TRUE genes. Prior to define metrics, let *G*_up_={*i*: gene *i* the expression of which is truly significantly induced} and *G*_down_={*i*: gene *i* the expression of which is truly significantly suppressed}.

#### Rank sum (RS)

We define the rank sum (RS) of TRUE genes as follows:

$$ \mathrm{RS}={\sum}_{i\in {G}_{up}\cup {G}_{down}}{\sum}_{j:{d}_i{d}_j>0}\mathrm{I}\left(\left|{d}_i\right|\le \left|{d}_j\right|\right) $$

where I(∙) is an indicator function. The reason for determining the ranks of genes with high and low expression is that the SAM procedure uses such a method when detecting genes of the two groups. We use the absolute value of test statistics because test statistics of genes with suppressed expression have negative values. For RS, lower values indicate better performance.

#### Top-ranked frequency (TRF)

The top-ranked frequency (TRF) of TRUE genes is computed by

$$ \mathrm{TRF}(r)=\#\left\{i\in {G}_{\mathrm{up}}\cup {G}_{\mathrm{down}}:{\sum}_{j:{d}_i{d}_j>0}\mathrm{I}\left(\left|{d}_i\right|\le \left|{d}_j\right|\right)\le r\right\}. $$

Here, *r* denotes the rank cutoff and is set to be smaller than the number of observations in *G*_up_ and *G*_down_. For a given cutoff *r*, TRF computes the number of TRUE genes ranked within *r*. For TRF, higher values indicate better performance.

To understand the performance metrics better, let us consider the following case. We have 100 genes and 10 TRUE genes among them. Assume that we obtain a top-ranked gene list as shown in Table 2 by a gene selection method. Among the 15 genes in the table, five are false genes (3^rd^, 7^th^, 8^th^, 12^th^, and 14^th^ genes in the table). In this case, RS = 76, TRF(5) = 4, and TRF(10) = 7.

**Table 2:** An example list of top-ranked genes

Gene rank	Rank of true genes	True or false
1	1	T
2	2	T
3	-	F
4	4	T
5	5	T
6	6	T
7	-	F
8	-	F
9	9	T
10	10	T
11	11	T
12	-	F
13	13	T
14	-	F
15	15	T
Rank sum	76	

## Results

### Simulation studies

**Fig. 4:**
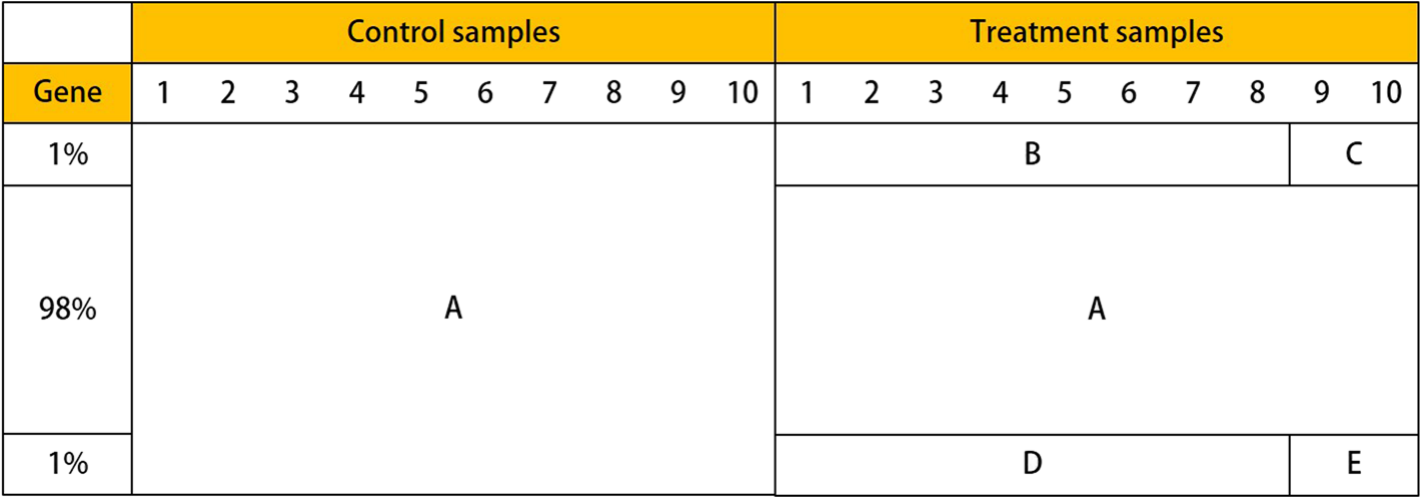
An example of simulated data structure. Each row and each column of this data frame correspond to a gene and a replicate sample, respectively, so we have a 10,000 × 20 data matrix in this study. We assume that there are 2% target genes (1% up-regulated and 1% down-regulated genes) among the total of 10,000 genes, and ten replicates in each group. There are five different distributions of data points: A, B, C, D, and E; groups C and E indicate outlier samples

**Fig. 5:**
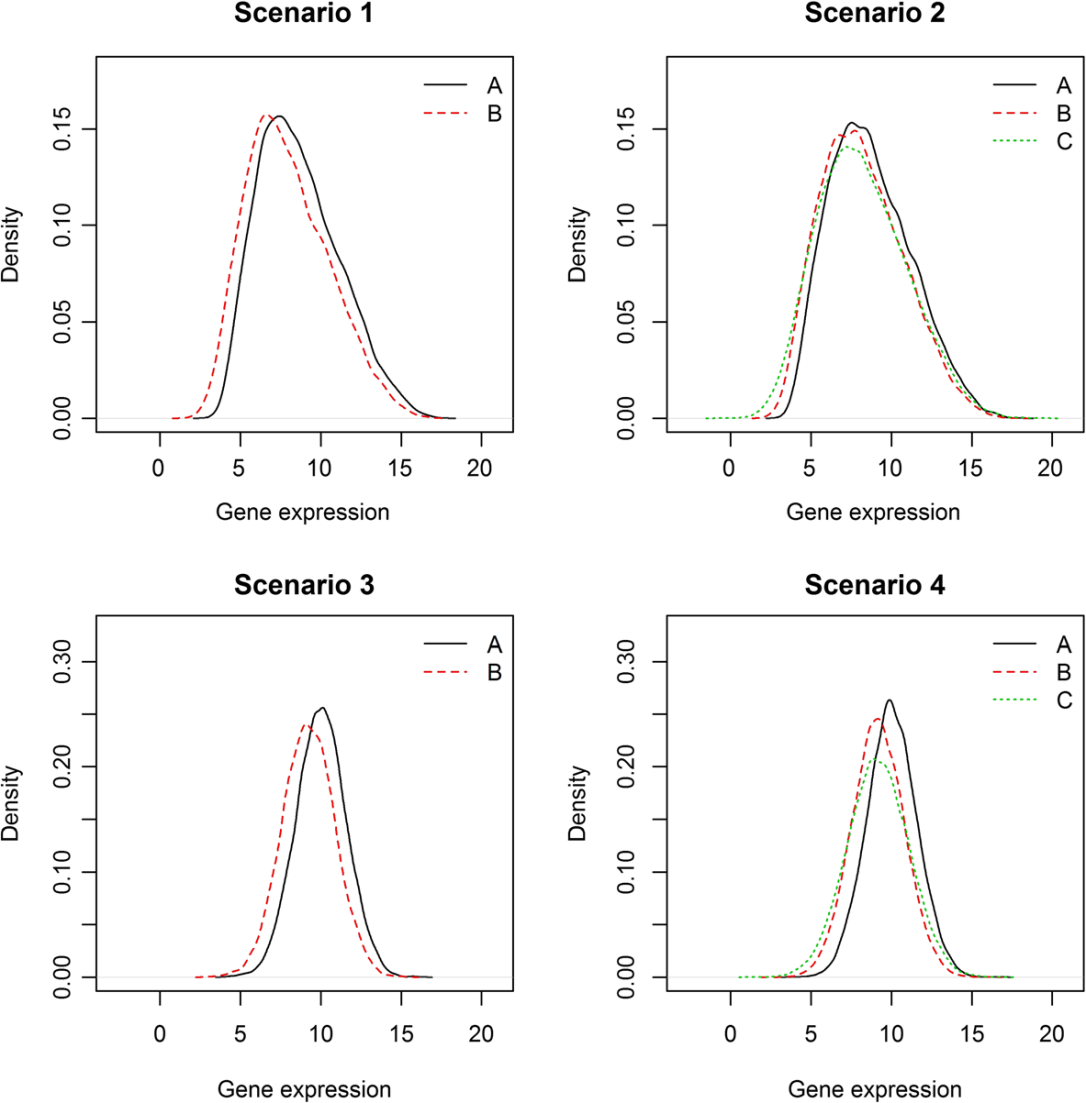
Empirical density of data points for scenarios 1, 2, 3, and 4. The solid line (**a**) for each plot is the density of control samples for target genes (**a**). The red dashed line (**b**) and green dotted line (**c**) are the densities of treatment samples for target genes. There are no green dotted lines (**c**) in the top-left and top-right plots because there are no outliers in scenarios 1 and 3

In this section, we compare gene selection methods using synthetic datasets. We consider four scenarios described above. For each scenario, we consider 7 different combinations of *n*_1_ and *n*_2_ in order to take into account the affects of sample size and class imbalance on gene selection performance as follows: (*n*_1_, *n*_2_) = (5, 5) , (5, 10) , (10, 5) , (10, 10) , (10, 15) , (15, 10) and (15, 15). For all scenarios, we assume that there are 2% target genes (1% up-regulated and 1% down-regulated genes) among the total of 10,000 genes. For simplicity, let us assume that the first 100 genes are downregulated and last 100 genes are upregulated. Then, we can describe the structure of our simulation data as shown in Fig. 4. This example illustrates the structure of noisy data containing outliers. In this case, the last two samples are outlying samples among 10 treatment samples of 200 target genes. There are five different distributions of data points: A, B, C, D, and E. For 9800 nontarget genes, the distributions of the control and treatment samples are the same (A). The first 100 downregulated genes are generated from two distributions (B and C) and the last 100 upregulated genes are also generated from two distributions (D and E). Groups C and E indicate outlier samples. If there are no outliers in the dataset, B is equivalent to C and D is equivalent to E. The empirical density plot of each group is shown in Fig. 5. For visualization, we use 5000 data points to ensure equivalent density of the points for each group (A, B, and C), that is, with a 1:1:1 ratio, not using the original ratio among the three groups.

**Fig. 6:**
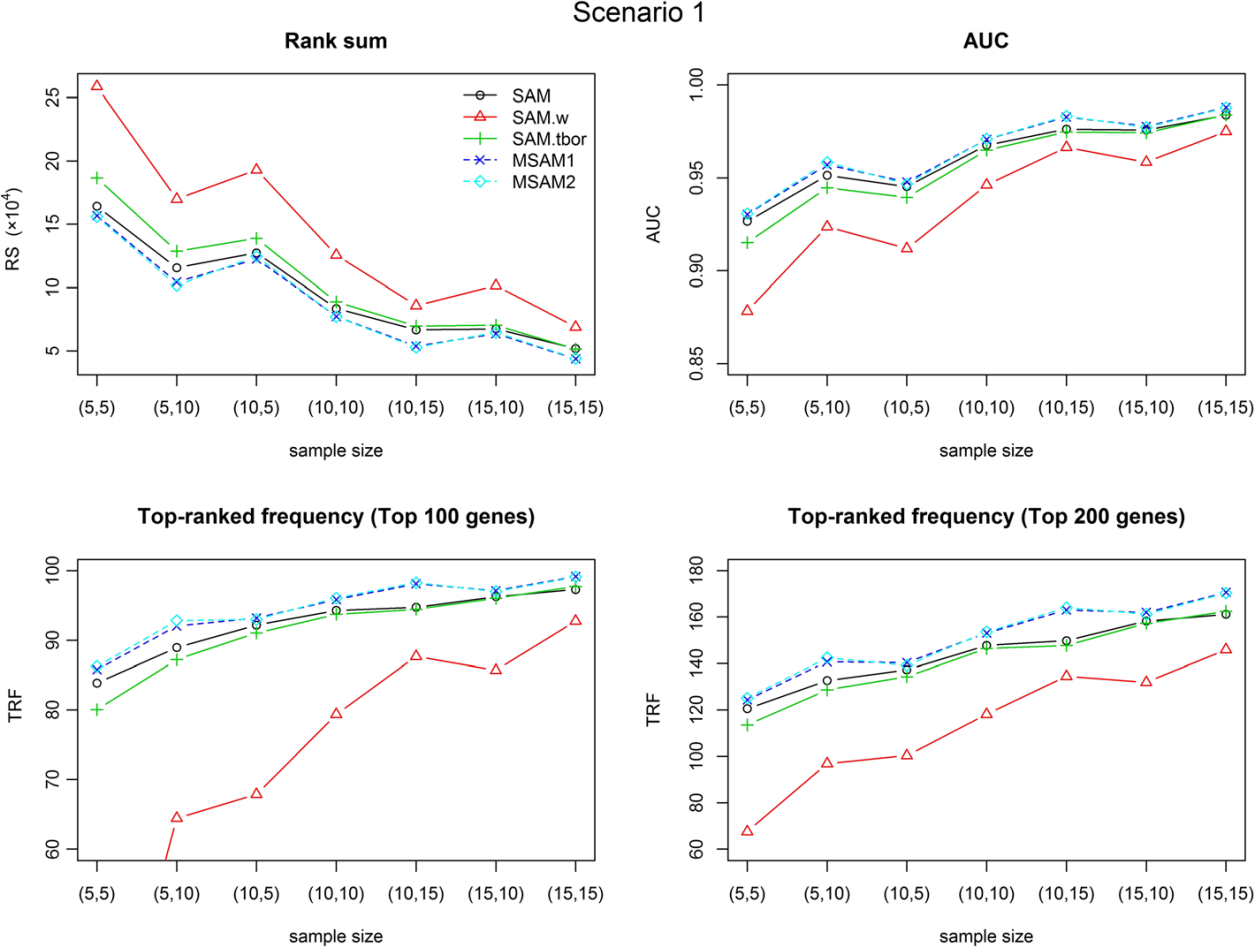
Simulation results for Scenario 1. Three solid lines (black, red, and green) indicate the results of three versions of SAM. Two dashed lines (blue and cyan) indicate the results of two versions of modified SAM. For RS, lower values are better. For AUC and TRF, higher values are better

**Fig. 7:**
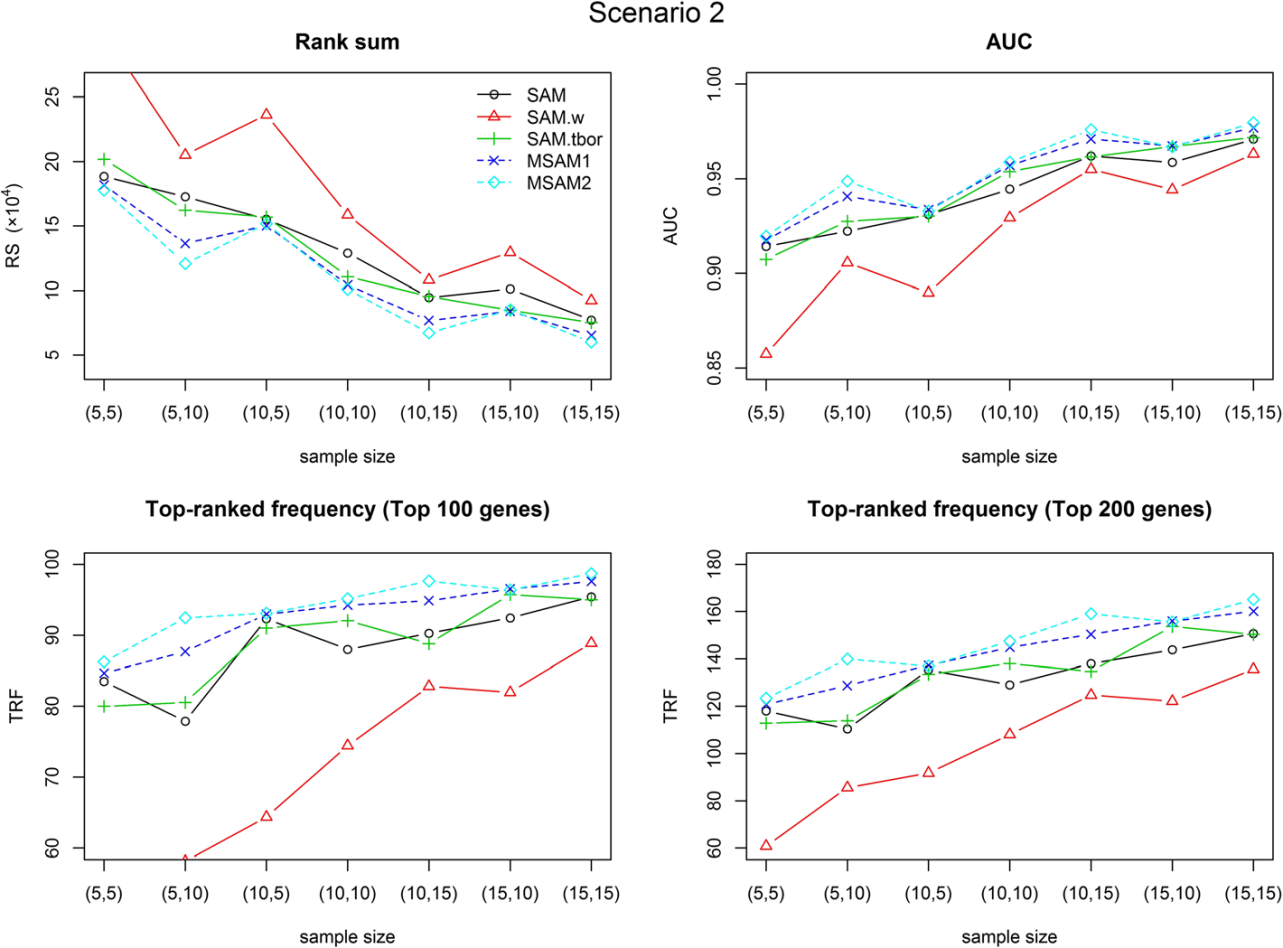
Simulation results for Scenario 2

We conduct simulation studies using synthetic data and compare the results using three metrics; two of them are RS and TRF, which were defined above, and the third is AUC. AUC is the area under a receiver operating characteristic (ROC) curve. Therefore, this value falls between 0 and 1, and higher values indicate better performance. We consider five gene selection methods, named SAM, SAM-wilcoxon, SAM-tbor, MSAM1 and MSAM2. SAM-wilcoxon is the Wilcoxon version of SAM [20, 32]. SAM-tbor is basically the same with SAM, except for applying a simple trim-based outlier removing algorithm to data prior to running SAM. In this study, we remove the largest and smallest observations from each sample type. Figs. 6 and 7 display the average performance of 100 simulations for each method on the three metrics. Table 3 shows numerical results of 4 cases. The best performance on each metric is shown in boldface. In scenario 1, the original SAM always outperform SAM-wilcoxon and SAM-tbor. Although SAM-tbor show better performance than SAM in some cases of scenario 2, its performance is worse than those of MSAMs. As can be seen from the figures and table, our proposed methods show better performance than three versions of SAM in all cases. In particular, modified SAMs are much better when given data is noisy (scenario 2, compared to scenario 1) and is a little better for less noisy cases. We can also see that our methods show more robust performance in all cases. When there is two outliers among ten samples, the number of target genes found by original SAM is reduced by 2–17%, whereas that found by MSAMs is reduced by 1–8%. In particular, when *n*_1_ = 5, *n*_1_ = 10 in scenario 2, SAM fails to detect 90 genes among the 200 TRUE genes, whereas MSAM2 fails to detect only 60 genes on average. Simulation results of scenarios 3 and 4 are in Additional file 1. These results are very similar with those of scenarios 1 and 2; MSAMs always perform better than three versions of SAM.

**Table 3:** Simulation results for 4 cases

	Scenario 1, *n*_1_ = 5, *n*_2_ = 10				Scenario 1, *n*_1_ = 10, *n*_2_ = 10			
	RS	AUC	TRF		RS	AUC	TRF	
Rank cutoff			100	200			100	200
SAM	115,542	0.95	88.93	132.67	83,544	**0.97**	94.28	147.74
SAM-w	169,865	0.92	64.43	96.84	125,513	0.95	79.37	118.18
SAM-tbor	128,588	0.94	87.21	128.67	88,765	0.96	93.72	146.42
MSAM1	104,236	**0.96**	92.02	140.70	77,317	**0.97**	95.84	153.08
MSAM2	**101,705**	**0.96**	**92.81**	**142.47**	**77,109**	**0.97**	**96.05**	**153.58**
	Scenario 2, *n*_1_ = 5, *n*_2_ = 10				Scenario 2, *n*_1_ = 10, *n*_2_ = 10			
	RS	AUC	TRF		RS	AUC	TRF	
Rank cutoff			100	200			100	200
SAM	172,669	0.92	77.88	110.38	128,966	0.94	87.97	129.06
SAM-w	205,161	0.91	58.14	85.55	158,618	0.93	74.44	108.06
SAM-tbor	162,252	0.93	80.54	113.94	110,655	0.95	92.05	137.97
MSAM1	136,442	0.94	87.69	128.76	104,286	**0.96**	94.23	144.82
MSAM2	**120,594**	**0.95**	**92.45**	**139.89**	**100,887**	**0.96**	**95.15**	**147.48**

Note: the best performance in each case is shown in **bold type**

### Real data analysis 1: *Fusarium*

The *Fusarium* dataset contains 17,772 genes and nine samples: three each from control, dtri6, and dtri10 groups [28]. Robust multichip analysis algorithm is used for condensing the data for the following [33]: extraction of the intensity measure from the probe level data, background adjustments, and normalization. The post-processed dataset used in [28] are stored at PLEXdb (http://www.plexdb.org) (accession number: FG11) [26]. As this data was from gene mutation experiments, researchers provided a list of genes that are differentially expressed between control and treatment (dtri6, dtri10) groups. These genes are as follows: fgd159-500_at (conserved hypothetical protein), fgd159-520_at (trichothecene 15-O-acetyltransferase), fgd159-540_at (Tri6 trichothecene biosynthesis positive transcription factor), fgd159-550_at (TRI5_GIBZE – trichodiene synthase), fgd159-560_at, fgd159-600_at (putative trichothecene biosynthesis), fgd321-60_at (trichothecene 3-O-acetyltransferase), fgd4-170_at (cytochrome P450 monooxygenase), fgd457-670_at (TRI15 – putative transcription factor), fg03534_s_at (trichothecene 15-O-acetyltransferase), fg03539_at (TRI9 – putative trichothecene biosynthesis gene), and fg03540_s_at (TRI11 – isotrichodermin C-15 hydroxylase).

In real data analysis sections, we only consider SAM, MSAM1, and MSAM2, all of which show good performance in simulation studies; we found that SAM-wilcoxon and SAM-tbor are worse than the original SAM in the previous section. Moreover, we cannot apply SAM-tbor to this data because this data has only three sample replicates in each group. Like this case, we can see that such a trim-based method is limited in its applications.

Tables 4 and 5 show the rank of 11 reference genes that are differentially expressed between the control group and the treatment groups (dtri6 and dtri10, respectively). The last row in each table indicates the rank sum of these 11 genes. As we can see, MSAM2 shows the best performance because the rank sum of this method is the smallest among those of the three gene selection methods. In particular, MSAMs improve the rank of the genes named fgd4-170_at and fgd159-500_at. For each of these genes, the result for one of their treatment samples is far from those for the other two samples. From the analysis, it can be asserted that our proposed methods efficiently identify the genes whose replicate samples contain an outlier, such as fgd4-170_at and fgd159-500_at.

**Table 4:** Rank of genes of interest: control versus dtri6

		SAM		MSAM1		MSAM2	
Gene	${\overline{x}}_i-{\overline{y}}_i$	${\overset{\sim }{d}}_i$	rank	${\overset{\sim }{d}}_i$	rank	${\overset{\sim }{d}}_i$	rank
fgd457-670_at	−4.82	−25.45	1	−19.78	1	−8.62	3
fgd159-550_at	−5.13	−24.58	2	−19.20	2	−9.27	2
fgd159-600_at	−5.38	−18.24	6	−14.10	6	−8.12	4
fg03534_s_at	−4.48	−14.70	7	−10.29	7	−5.64	7
fg03540_s_at	−3.34	−13.56	8	−9.97	9	−5.02	14
fgd321-60_at	−3.71	−13.26	9	−9.50	10	−5.28	10
fg03539_at	−3.80	−13.21	10	−10.07	8	−5.19	12
fgd159-500_at	−3.66	−12.49	11	−9.01	11	−5.39	9
fgd159-520_at	−5.08	−11.60	12	−8.70	12	−5.62	8
fgd159-540_at	−4.06	−10.73	18	−8.59	13	−5.03	13
fgd4-170_at	−4.98	−9.22	26	−7.58	21	−5.21	11
Rank sum			110		100		**93**

Note: the best performance in terms of rank sum is shown in **bold type**

**Table 5:** Rank of interest genes: control versus dtri10

		SAM		MSAM1		MSAM2	
Gene	${\overline{x}}_i-{\overline{y}}_i$	${\overset{\sim }{d}}_i$	rank	${\overset{\sim }{d}}_i$	rank	${\overset{\sim }{d}}_i$	rank
fg03539_at	−6.66	−22.18	1	−17.27	1	−10.46	2
fg03534_s_at	−4.00	−11.24	4	−8.42	4	−5.68	4
fgd159-560_at	−2.76	−9.74	5	−8.01	5	−5.22	6
fgd159-600_at	−3.28	−8.77	8	−6.81	8	−4.92	7
fgd159-520_at	−4.17	−8.12	9	−6.31	10	−4.65	8
fgd457-670_at	−3.35	−6.44	16	−5.05	18	−3.83	12
fgd4-170_at	−3.53	−5.73	21	−4.76	19	−3.68	13
fgd159-550_at	−3.22	−4.89	26	−4.23	22	−3.42	16
fg03540_s_at	−2.35	−4.84	27	−3.88	27	−2.90	23
fgd321-60_at	−1.62	−3.07	60	−2.44	64	−1.88	38
fgd159-500_at	−1.73	−2.88	78	−2.37	73	−1.90	37
Rank sum			255		251		**166**

Note: the best performance in terms of rank sum is shown in **bold type**

**Fig. 8:**
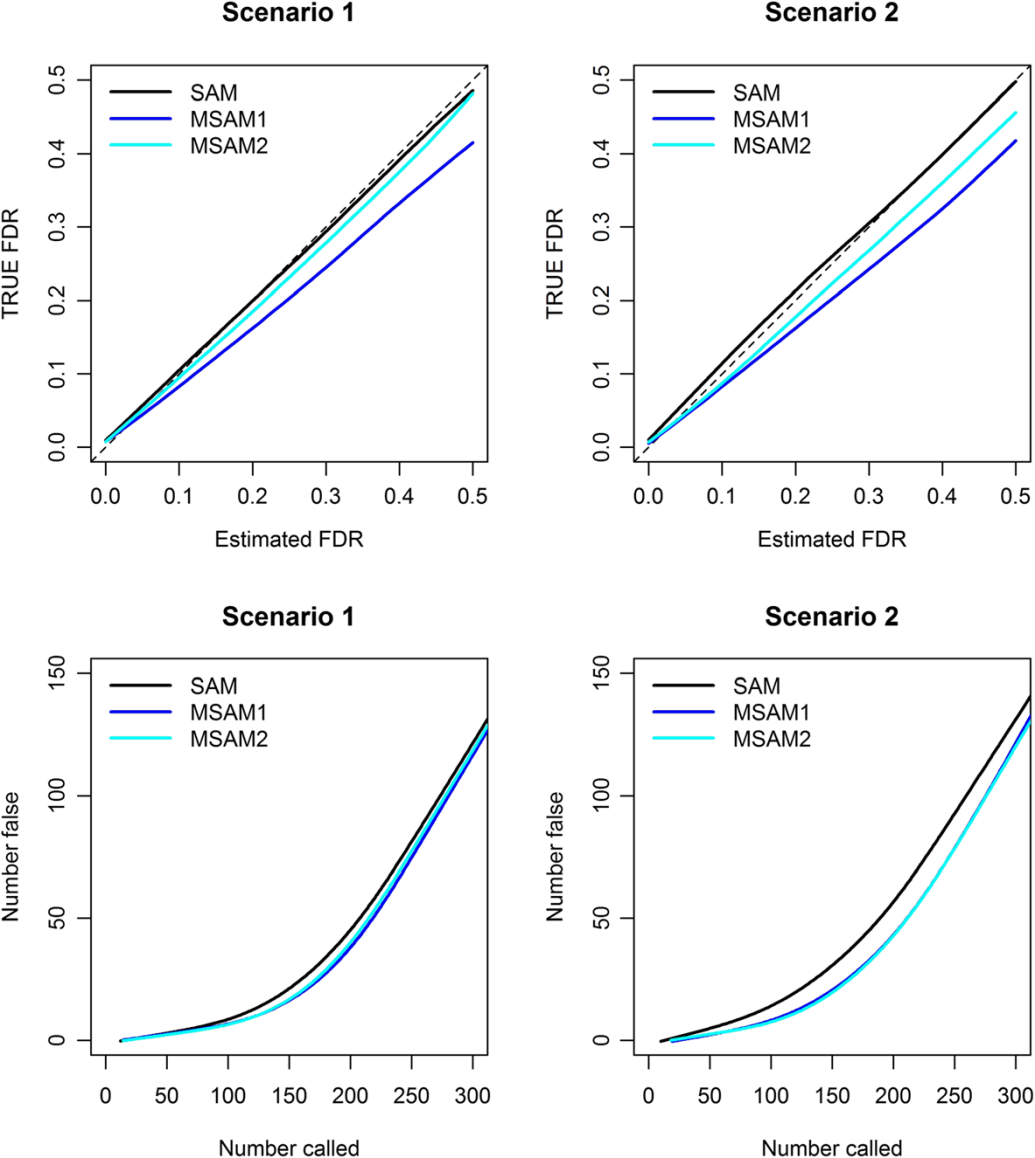
The top two plots show TRUE FDR vs. estimated FDR and the bottom two plots show the number of falsely detected genes relative to the total number of detected genes for scenario 1 and 2. In each top plot, the solid lines indicate estimation curves of each method and the dashed line represents ***Y*** = ***X***

### Real data analysis 2: Leukemia

Leukemia is a cancer of the bone marrow, where blood cells are made. In leukemia, abnormal blood cells are produced in the bone marrow and crowd out other normal blood cells. Depending on the type of abnormal blood cells that are multiplying, leukemia can be classified as acute lymphocytic leukemia (ALL) or acute myeloid leukemia (AML). Identifying the type of leukemia is very important because patients should receive different treatments according to the disease type. [29] studied a generic approach to cancer classification based on gene expression and provided a list of 50 significant genes for classifying ALL and AML. After this study, this dataset has been widely used in transcriptomic analysis, e.g., [34, 35]. This data are available in the *golubEsets* library in Bioconductor [27]. The original data consist of 38 samples (27 from ALL patients and 11 from AML patients) and 7129 genes. We randomly selected five, seven, and ten samples for each sample type and repeated this experiment 100 times for averaging because biological experiments usually have a small number of samples owing to limitations of time and resources. It is thus important that a method shows good performance even if the sample size is small.

The simulation results are shown in Table 6. In this table, RS and TRF values of three gene selection methods, which were computed by using 50 genes that are considered informative in [29] over 100 trials. For each case, the best performance is shown in boldface in the table. As we can see, MSAM1 or MSAM2 performs better than SAM in terms of RS and TRF, regardless of rank cutoff values. The overall performance of SAM and MSAM1 are very similar, but MSAM1 always performs slightly better than SAM. In the point of view of sample size, MSAM2 outperform SAM and MSAM1 when the sample size is very small, e.g., 5, and MSAM1 performs better than SAM and MSAM2 when the sample size is moderate, e.g., 7 and 10. As the sample size increases, all of the three methods identify informative genes better.

**Table 6:** Rank sum and top-ranked frequency of informative genes in Leukemia data

	# picked samples: 5			# picked samples: 7			# picked samples: 10		
				Rank sum					
	SAM	MSAM1	MSAM2	SAM	MSAM1	MSAM2	SAM	MSAM1	MSAM2
	22,287	21,585	**15,815**	11,924	**11,790**	13,286	5566	**5534**	11,256
				Top-ranked frequency					
*r*	SAM	MSAM1	MSAM2	SAM	MSAM1	MSAM2	SAM	MSAM1	MSAM2
20	5.05	5.29	**7.46**	7.56	7.86	**9.05**	11.93	**12.15**	10.34
40	7.74	8.24	**12.72**	12.55	12.95	**14.48**	19.11	**20.05**	16.70
60	10.30	11.19	**16.46**	16.33	17.09	**18.02**	25.10	**25.89**	20.23
80	12.76	13.54	**19.02**	19.78	20.52	**20.63**	29.30	**30.27**	22.71
100	14.67	15.74	**21.36**	22.59	**23.52**	23.07	32.72	**33.54**	24.75
120	16.72	17.75	**23.43**	24.76	**25.74**	25.45	35.65	**36.22**	26.94
140	18.38	19.48	**25.25**	26.90	**27.67**	27.29	37.77	**38.16**	28.91
160	20.03	20.94	**26.79**	28.67	**29.74**	28.92	39.60	**39.92**	30.48

Note: the best performance for each rank cutoff is shown in **bold type**

### FDR comparison

In this section, we discuss the FDR estimation procedures of SAM, MSAM1, and MSAM2. FDR is used in SAM procedure in order to deal with a multiple testing problem. The SAM interface in R, *samr* package [20], provides a significant gene list based on the FDR value that is estimated by its internal function. We also construct our own interface for MSAMs in R, based on the *samr* package, in order to allow for users to apply our proposed methods to their transcriptome research; see Additional file 2. Users start the procedure by setting their desired FDR value (for example, 0.2). We will call this value ‘estimated FDR’. Based on the estimated FDR, our procedure calculates the value of corresponding *Δ* and identifies potentially significant genes. In real applications, we do not know TRUE FDR, so the estimated FDR is used as a substitute for TRUE FDR. If the estimated value is different from the true value, the number of genes that are detected using the estimated FDR is larger or smaller than the true number. Therefore, users may be interested in how well SAM and MSAMs procedures estimate TRUE FDR value. To this end, in this section, we evaluate SAM, MSAM1, and MSAM2, focusing on their FDR estimation performances.

Since we know the number of TRUE significant genes in our simulated datasets, we can compare the estimated FDR and TRUE FDR in simulation study. After 100 simulations, we draw a scatter plot of the TRUE FDR versus the estimated FDR by calculating the average values of the TRUE FDR for each estimated FDR. We next draw a smooth curve close to the scatter plot for scenarios 1 and 2 to find the estimation accuracy at various levels of FDR. In particular, the estimation accuracy at low FDR is important since researchers generally set FDR at a small value so as to avoid having a large proportion of falsely significant genes among the detected genes. For this reason, we only show the results when the estimated FDR is lower than 0.5. Figure 8 displays the results; see the top two plots. As we can see, SAM estimates the TRUE FDR very accurately and two modified SAMs slightly overestimate the TRUE FDR. In other words, our methods have conservative property in their FDR estimation. However, the conservative estimation of FDR may not cause serious problems for the analysis when we use FDR as an upper bound of a tolerable error [36].

For such an analysis, the more important thing is how many non-significant genes are included in the detected genes. Because the truths are known in the simulated data, we can calculate the number of falsely detected genes among the identified genes. With the same number of total positives, the method with the smallest number of false positives is the best [36]. Using the plotting method described above, a smooth curve of the number of false positive genes versus the total number of identified genes are drawn. Figure 8 shows the results From the figure, we can see that MSAM1 and MSAM2 gives smaller number of false positive genes than SAM across all noise level and the total number of identified genes. From the results, we can say that MSAMs are better than SAM because they includes the less number of false genes in the selected gene subset.

When we estimate FDR, we calculate both median FDR and mean FDR to determine which estimate more closely approximates the true value. Since the original *samr* interface provides the median FDR and 90th percentile FDR only, we modified its estimation function and obtained the median and mean values of FDR. As a result, we found that the median FDR was closer than the mean FDR to the TRUE FDR for all methods. This coincides with results published elsewhere [37], in which the median FDR was recommended as a criterion for gene selection methods when the estimated proportion of differentially expressed genes is greater than 1%, regardless of the sample size. Based on these results, we use the median value instead of the mean value when estimating FDR.

### Classification analysis

Once important genes are identified from thousands of genes, they can be used to predict two different experimental states or responses (for example, cancer and normal). Therefore, we also examine how well a few top genes selected by each method identify the true classes. We attach these results in Additional file 3. In this file, we introduce 4 datasets we used and explain the construction of classifiers, 6 gene selection methods, 3 performance metrics to be considered in this study. Our comments on the results are also included. As can be seen in the file, our proposed methods, MSAMs, show quite good performances in all cases. In this additional section, we prove their competitiveness in classification tasks, not only in gene selection tasks.

## Discussion

In transcriptome data analysis, most studies have been devoted to developing filter-based methods that are the simplest and fastest, and most computationally efficient. Hybrid methods, which are generally the combination of filter and wrapper methods, have recently gained popularity in the literature [13]. These methods consist of two steps: First, relevant features are selected by a filter method and the remaining features are eliminated. Second, a wrapper method verify these features and determine the final feature set that gives high classification accuracy [16]. In this point of view, filter methods have a lot of flexibility as they can be combined with not only any learning algorithm, but also any gene selection method, such as a wrapper method, resulting in a hybrid method. The performance of a hybrid method relies totally on the combination of filter and wrapper methods as well as the classifier [18]. We believe that accurate gene selection by filter methods clearly allow better classification accuracy. Therefore, our new filter-based methods will be useful not only in gene selection, but also constructing a good classifier in microarray applications.

Our experiments showed the efficiency of our methods; it was demonstrated that when the same number of genes were selected, our methods included the less number of false genes than the conventional method. Our results also strongly suggest that these newly proposed methods outperform the conventional method and show quite consistent performance, even with a high noise level and a small sample size. Given that noisy data and a small sample size are commonly encountered in microarray studies [30, 38–40], we believe that our methods will prove useful.

This research was based on the existing interface of SAM that was modified to apply our proposed methods. This modified version of the *samr* package is available in Additional file 2. We attempted to find a balance between flexibility and control in the usage of our methods by allowing users to set particular parameters and by minimizing the number of modifications to the original interface. Additional file 2 includes a detailed explanation of what we changed, but users can easily apply our methods to their own datasets without reading the manuscript in the first file, since we provide some simple and useful examples of detecting differentially expressed genes using our methods in Additional file 4. We also provide two real datasets and one simulated dataset used in this study (see Additional files 5, 6 and 7). All of the additional files are also available at author’s homepage (http://home.ewha.ac.kr/~josong/MSAM/index.html).

## Conclusions

We have proposed new test methods for identifying genes that are differentially expressed between two groups in microarray data and evaluated their performance using a series of simulated data and two real datasets. The results have demonstrated that our proposed methods identified target genes better than the original method, SAM, for both simulation studies and real data analysis. Using our weighting schemes, significant genes can be selected in a more robust manner by avoiding the overestimation of variance. In particular, these procedures are very effective when the given data are noisy or the sample size is limited. Therefore, they prevent technical or biological problems that can occur in biological experiments and data pre-processing from impeding accurate gene selection. We believe that our proposed methods can be applied to various datasets in other fields if they have characteristics similar to microarray data.

## Additional files

Additional file 1: Additional simulation results for scenario 3 and 4 (DOCX 399 kb)

Additional file 2: . R code for the modified *samr* package. (R 29 kb)

Additional file 3: Classification analysis section (DOCX 564 kb)

Additional file 4: R code for some examples of our method for detecting genes that are differentially expressed. (R 2 kb)

Additional file 5: Fusarium data (CSV 2107 kb)

Additional file 6: Leukemia data (CSV 1168 kb)

Additional file 7: Simulated data (scenario 2) (CSV 2380 kb)

## Abbreviations

ALL: acute lymphocytic leukemia
AML: acute myeloid leukemia
AUC: area under the curve
FDR: false discovery rate
MSAM1: modified SAM1
MSAM2: modified SAM2
ROC: receiver operating characteristic
RS: rank sum of true genes
SAM: significance analysis of microarrays
TRF: top-ranked frequency of true genes
