# Supplementary material for: Robust gene selection methods using weighting schemes for microarray data analysis
Source: BMC Bioinformatics. 2017 Sep 2;18:389. doi: 10.1186/s12859-017-1810-x (PMC5581932; doi:10.1186/s12859-017-1810-x)
Supplement: Supplementary file 1 — Additional simulation results for scenario 3 and 4 (DOCX 399 kb) [file 12859_2017_1810_MOESM1_ESM.docx]

**Additional File 1**

: This file provides additional simulation results (for scenario 3 and 4) of “Robust gene selection methods using weighting schemes for microarray data analysis”.


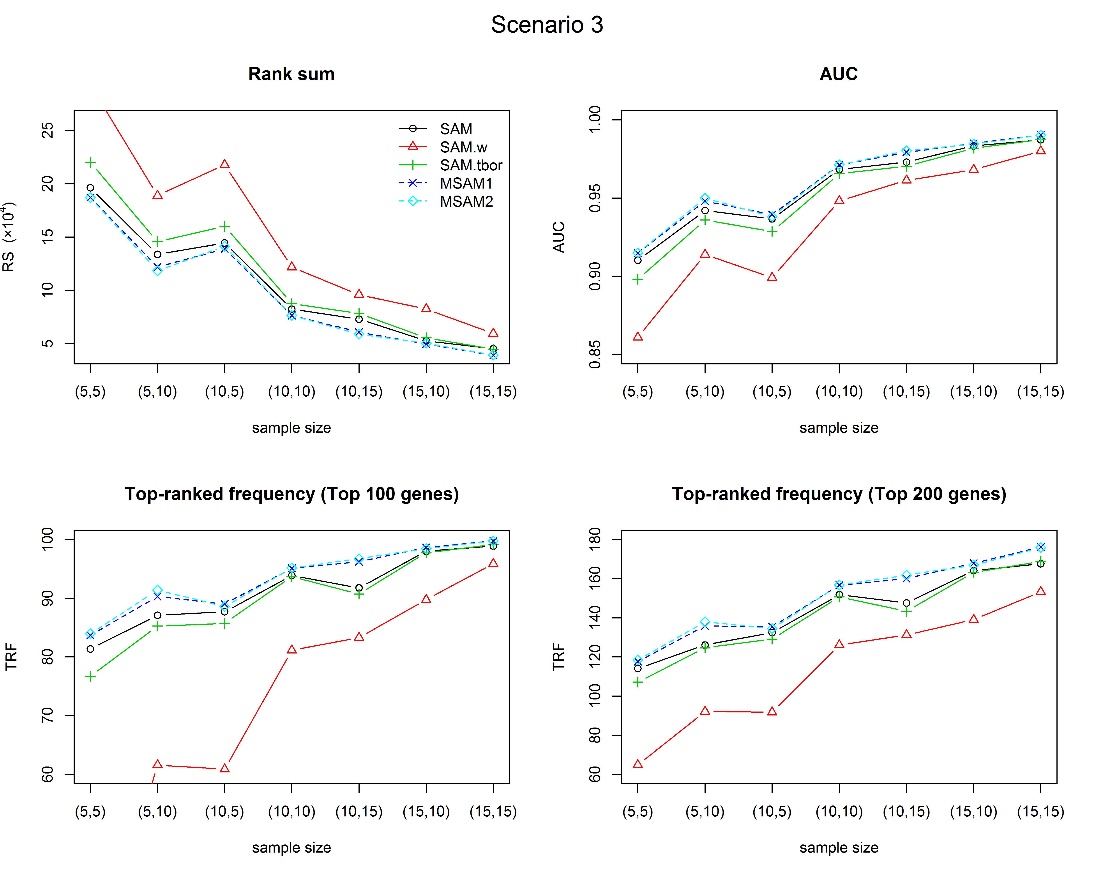


Figure S1. Simulation results for scenario 3.


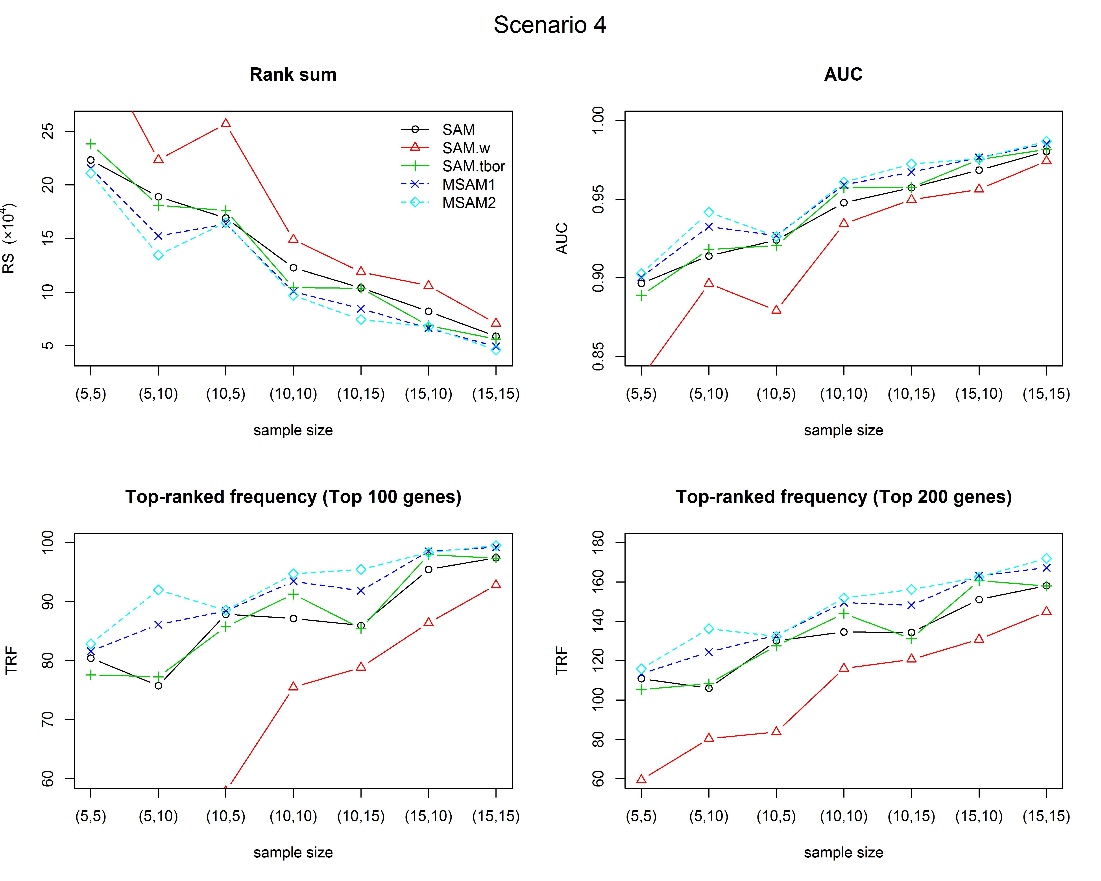


Figure S2. Simulation results for scenario 4.

As we can see from these two figures, MSAMs performs better than three versions of SAM in all cases.
