# Supplementary material for: Robust gene selection methods using weighting schemes for microarray data analysis
Source: BMC Bioinformatics. 2017 Sep 2;18:389. doi: 10.1186/s12859-017-1810-x (PMC5581932; doi:10.1186/s12859-017-1810-x)
Supplement: Supplementary file 3 — Classification analysis section (DOCX 564 kb) [file 12859_2017_1810_MOESM3_ESM.docx]

**Additional File 3**

: This file provides classification analysis section of “Robust gene selection methods using weighting schemes for microarray data analysis”.

There are many studies about the classification analysis for microarray data [1-4]. We selected 4 well-known binary problems [5-8] from the studies and considered 6 gene selection methods that achieved good classification accuracy. **Table S1** shows the brief description of the datasets we used. From the previous studies, we found that two important factors for classification tasks in microarray data are gene selection method and classifier. Details for our gene selection and classifier construction method are described below. Since the classification performance of each method is extremely varying from data to data, we here report our results in individual dataset and then show combined results in order to find their overall performances across all of the datasets.

Table S1. Summary of datasets used in classification analysis.

| Dataset | Reference | Classes | # samples | # genes |
| --- | --- | --- | --- | --- |
| DLBCL | [6] | Follicular or Germinal | 58/19 | 7129 |
| Prostate | [7] | Prostate or non-Prostate | 50/52 | 12625 |
| Colon | [5] | Tumor or Normal | 40/22 | 2000 |
| Myeloma | [8] | Presence or Absence of focal lesions of bone | 36/137 | 12625 |

*Construction of classifiers*

Among many machine learning algorithms, support vector machine (SVM) has been popular for classification tasks in microarray data. For example, [1] demonstrated its robustness in analyzing microarray data. Furthermore, many comprehensive studies [3,9] has shown the SVM’s superiority over other machine learning algorithms across a number of well-known microarray problems. One of important issues for constructing a SVM classifier is kernel selection. In this study, we use a radial basis function (RBF) kernel, which is commonly used in many other applications. Generally, when dealing with high-dimensional feature space such as microarray platforms, linear and polynomial kernels are popular because we may not need to map the data into a higher dimensional space. In this research, however, we select a few genes and then construct a SVM classifier. So we decided to use the RBF kernel for nonlinear mapping. It is also reported that SVM classifiers with the RBF kernel show good performances in such analysis [3].

*Gene selection methods*

In order to compare our methods with many other techniques, we decided to include more gene selection methods in our classification analysis. Previously, [2] compared 10 gene selection methods using four supervised classifiers over 9 different binary (two class) microarray datasets; it was shown that Rank products (RP) [10] outperformed others at small sample size or high levels of noise and Empirical bayes [11] performed well across a range of sample sizes. Among them, RP is based on fold change (FC) information. After that, [4] proposed a new feature selection method, fold change rank ordering statistics (FCROS), which is also based on FC, and showed that FCROS was computationally faster and detected significant genes better than RP. Therefore, in this study, we compare SAM, SAM-wilcoxon, Empirical bayes, and FCROS with our two methods, MSAM1 and MSAM2. We call them SAM, SAM-w, EBayes, FCROS, MSAM1, and MSAM2 respectively in this article.

*Classification performance metrics*

From a binary classifier, there are 4 possible outcomes: true positives (TP), true negatives (TN), false positives (FP), and false negatives (FN). They are used to compute classification performance metrics. In this paper, we consider the following three performance metrics to compare above six gene selection methods: AUC, accuracy, and g-mean [12]. AUC is the area under a receiver operating characteristic (ROC) curve. Accuracy, which is the most popular metric, is the proportion of correctly classified samples among total samples: Accuracy = (TP+TN)/(TP+FP+TN+FN). G-mean is the geometric mean of sensitivity (i.e., true positive rate) and specificity (i.e., true negative rate) where sensitivity = TP/(FN+TP) and specificity = TN/(TN+FP). Therefore, g-mean can evaluate the performance of a classifier, taking into consideration class imbalance in data.

*Analysis procedure and results*

The overall classification procedure is as follows. We first divide the whole data into 70% training set and 30% test set. Samples in the test set are treated as unknown samples and be used to estimate prediction accuracy. With the training set, we score all the genes using the six gene selection methods. Each gene selection method may provide different gene rankings. Then, we create new datasets consisting of the top 20, 40, 60, 80, and 100 genes and construct SVM classifiers using the data. As the RBF kernel of the SVM classifier has two parameters, we use 10-fold cross validation to find their optimal values and use the optimized values when constructing the classifier. With the test set, 3 types of test errors (AUC, accuracy, and g-mean) are recorded and the whole procedure is repeated 100 times for averaging. This procedure is illustrated in **Figure S3**.


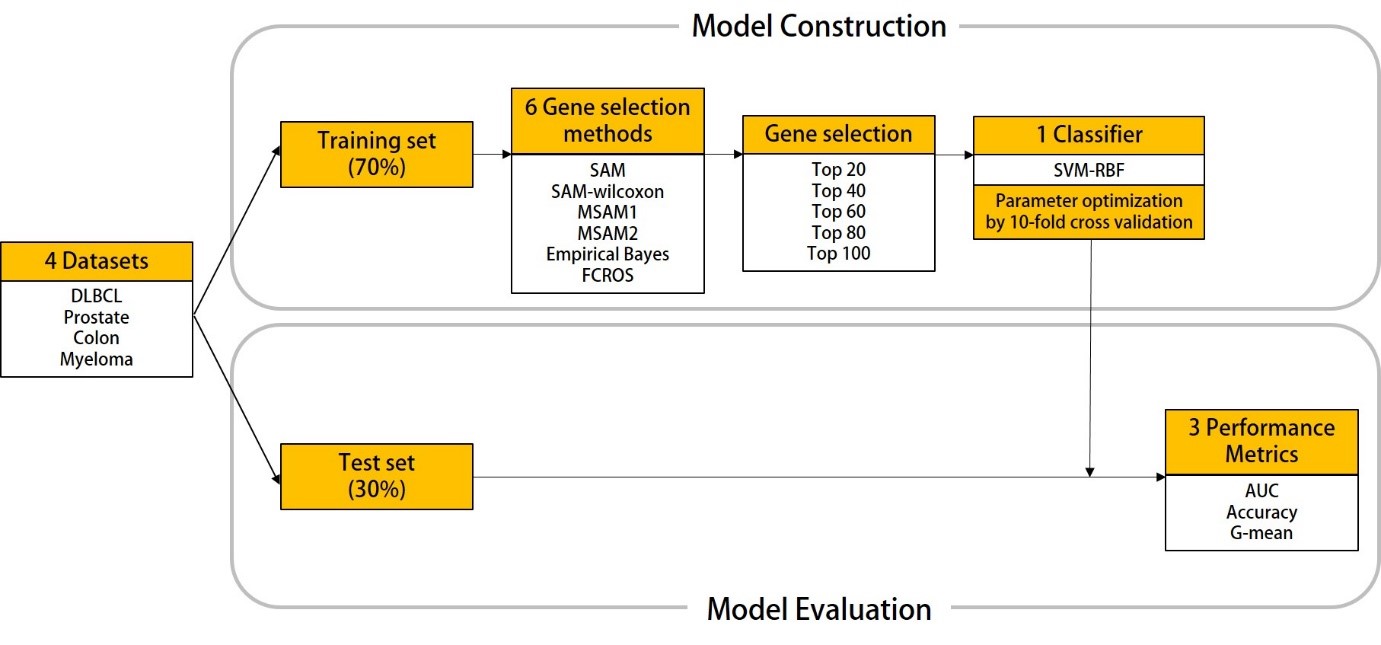


Figure S3. Method overview of our classification analysis.

From **Figure S4**, we can find the classification performances of 6 gene selection methods in each dataset. It seems that although all the methods tend to achieve better classification accuracy as the number of selected genes are greater, the performance difference is not much and they show good performance only with 20 genes. In particular, MSAMs and EBayes show good performances in highly imbalanced data (Myeloma). SAM shows relatively good performance in nearly balanced data (Prostate). The performance of EBayes is varying from data to data; it shows the best performances in only one dataset, and shows the worst or nearly worst classification accuracy in other datasets. To find the overall classification performance of each method, we compute the average ranks of 6 gene selection methods by combing their classification results from all cases (dataset and the number of selected genes). **Table S2** shows the results. In this table, smaller values are better; the average rank of the best method is close to 1 and that of the worst method is close to 6. As we can see from **Figure S4** and **Table S2**, MSAMs and FCROS is the best three methods among the 6 gene selection methods.

Table S2. Average rank of 6 gene selection methods over total 20 cases (4 real datasets and 5 gene subset sizes).

|  | AUC | Accuracy | G-mean |
| --- | --- | --- | --- |
| SAM | 3.45 | 3.70 | 3.70 |
| SAM-w | 4.05 | 4.65 | 4.50 |
| MSAM1 | 3.65 | 3.25 | 2.95 |
| MSAM2 | 2.60 | **2.15** | **2.25** |
| EBayes | 4.75 | 4.85 | 4.75 |
| FCROS | **2.50** | 2.40 | 2.85 |

Note: the best performance in each metric is shown in bold type.


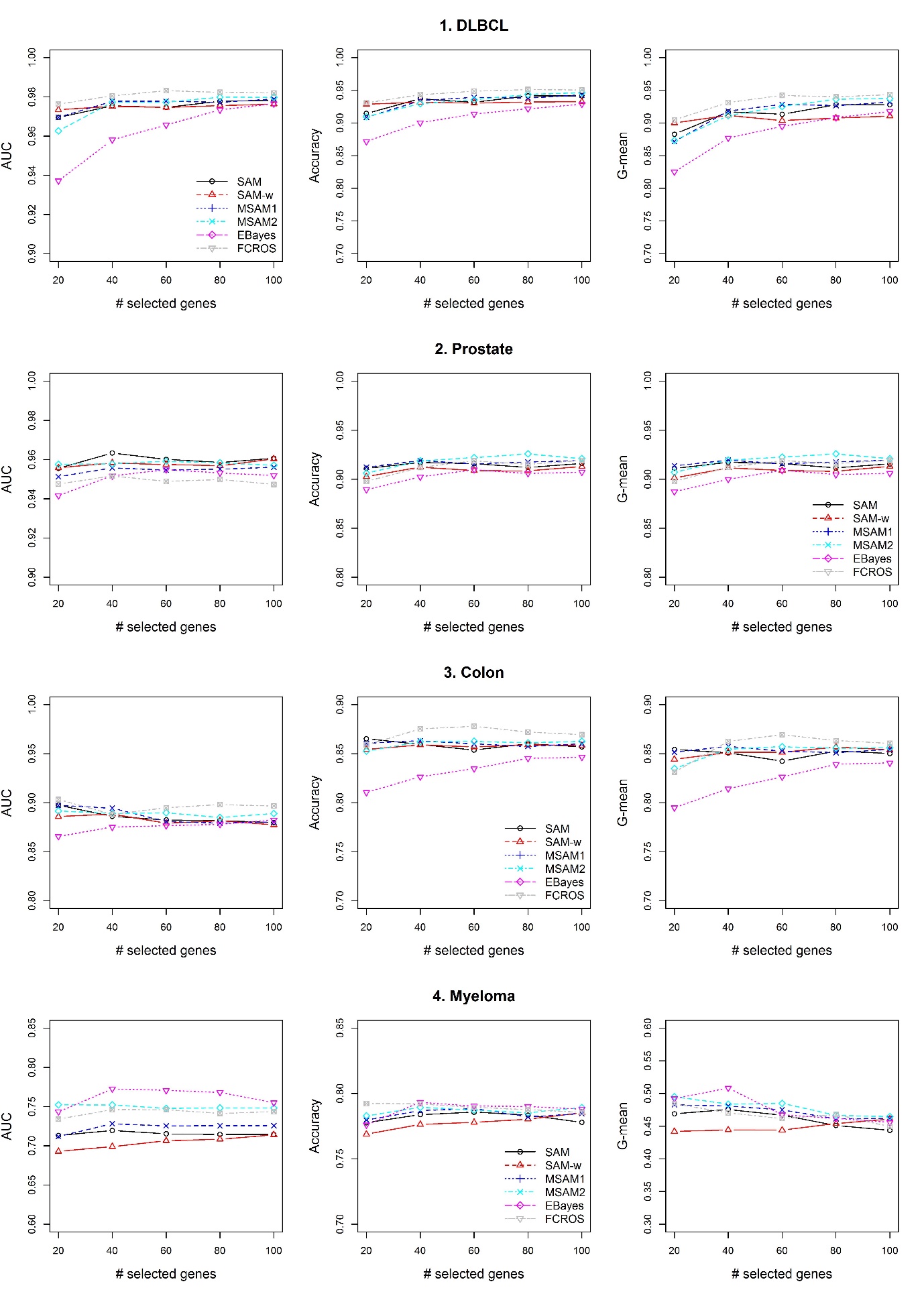


Figure S4. Classification performances of 6 gene selection methods relative to data and the number of selected genes.

**Reference**

1. Furey TS, Cristianini N, Duffy N, Bednarski DW, Schummer M, Haussler D. Support vector machine classification and validation of cancer tissue samples using microarray expression data. Bioinformatics. 2000;16(10):906-14.
2. Jeffery IB, Higgins DG, Culhane AC. Comparison and evaluation of methods for generating differentially expressed gene lists from microarray data. BMC bioinformatics. 2006;7(1):359.
3. Önskog J, Freyhult E, Landfors M, Rydén P, Hvidsten TR. Classification of microarrays; synergistic effects between normalization, gene selection and machine learning. BMC bioinformatics. 2011;12(1):390.
4. Dembélé D, Kastner P. Fold change rank ordering statistics: a new method for detecting differentially expressed genes. BMC bioinformatics. 2014;15(1):14.
5. Alon, U, Barkai N, Notterman DA, Gish K, Ybarra S, Mack D, Levine AJ. Broad patterns of gene expression revealed by clustering analysis of tumor and normal colon tissues probed by oligonucleotide arrays. Proc Natl Acad Sci. 1999;96(12):6745-50.
6. Shipp MA, Ross KN, Tamayo P, et al. Diffuse large B-cell lymphoma outcome prediction by gene-expression profiling and supervised machine learning. Nat Med. 2002;8(1):68-74.
7. Singh D, Febbo PG, Ross K, et al. Gene expression correlates of clinical prostate cancer behavior. Cancer Cell. 2002;1(2):203-9.
8. Tian E, Zhan F, Walker R, Rasmussen E, Ma Y, Barlogie B, Shaughnessy JD Jr. The role of the Wnt-signaling antagonist DKK1 in the development of osteolytic lesions in multiple myeloma. N Engl J Med. 2003;349(26):2483-94.
9. Brown MPS, Grundy WN, Lin D, Cristianini N, Sugnet CW, Furey TS, Ares M Jr, Haussler D. Knowledge-based analysis of microarray gene expression data by using support vector machines. Proc Natl Acad Sci USA. 2000;97(1):262-7.
10. Breitling R, Armengaud P, Amtmann A, Herzyk P. Rank products: a simple, yet powerful, new method to detect differentially regulated genes in replicated microarray experiments. FEBS Lett. 2004;573(1-3):83-92.
11. Efron B. Microarrays, empirical Bayes and the two-groups model. Stat Sci. 2008;23(1):1-22.
12. ///Kubat M, Matwin S. Addressing the curse of imbalanced training sets: one-sided selection. Proc 14th ICML. 1997;97:179-86.
